# The Predictive and Explanatory Power of the Contrast Theory of Myopia

**DOI:** 10.1167/tvst.14.3.11

**Published:** 2025-03-11

**Authors:** Jay Neitz, Maureen Neitz

**Affiliations:** 1 University of Washington, Department of Ophthalmology, Seattle, WA, USA. e-mail: jneitz@uw.edu

In their letter to the Editor regarding our published article,^1^ Gawne et al.^2^ suggest that our explanation of how diffusion optics technology (DOT) lenses work may be oversimplified. We proposed that DOT lenses reduce contrast and thereby decrease activity in peripheral midget bipolar cells, which in turn diminishes the drive for axial eye growth. DOT lenses were developed based on contrast theory, which is the most complete theory of myopia. It can be judged by its predictive and explanatory power. Visual signals originating from the peripheral retina locally influence scleral expansion during emmetropization. In primates, including humans, every cone in the peripheral retina connects to one ON- and one OFF-midget bipolar cell,^3^ which act as contrast detectors. Contrast theory holds that the activity of these cells drives axial growth of the eye. This predicts that lowering contrast reduces eye growth and myopia progression. It is the only theory that specifies precisely which post-receptoral neurons in the human retina carry the signals responsible for emmetropization. Moreover, contrast theory offers a potential signaling pathway from the bipolar cells to the back of the eye to control axial elongation. Bipolar cells make ectopic synapses onto dopaminergic amacrine cells, which release dopamine, a neurotransmitter implicated in regulating eye growth.^4^ In turn, these send processes toward the cone terminals where dopamine released may reach dopamine D2 receptors in the retinal pigment epithelium,^5^ whose activation may lead to axial elongation.

**Figure 1.**
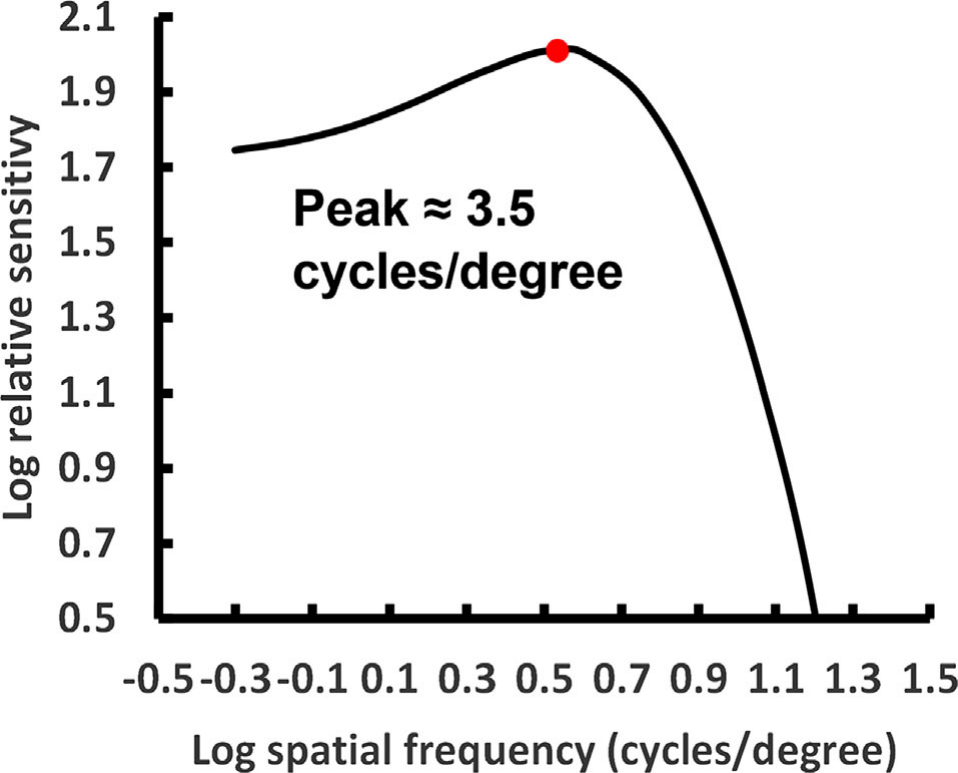
Difference of Gaussians model of the modulation transfer function of the human peripheral midget bipolar cells based on the cone spacing at 10° eccentricity^1^ and the modulation transfer function of the human eye.^6^

The encouraging news for advancing consensus in the myopia field is that there is substantial common ground between our perspective and the views Gawne and colleagues express in their letter to the Editor. We agree that other myopia control lenses that have proven effective, reduce peripheral image contrast, as do the DOT lenses. We also agree that reducing contrast is the key to myopia control and that the signaling pathway responsible for emmetropization is most sensitive to image data in the 3–4 cyc/deg range. However, we come to this latter conclusion not based on the animal studies referenced by Gawne et al.^2^ but rather on a difference-of-Gaussians model of the modulation transfer function of peripheral midget bipolar cells (Fig. 1) based on parameters from the optics of the eye.^6^ and the anatomy of the human retina at 10 degrees eccentricity.^3^^,^^7^ The agreement presumably derives from the similarities between animal vision and peripheral vision in humans.

The fact that contrast theory precisely specifies the response characteristics of the neurons in the human retina that carry the signals responsible for emmetropization has made it possible to design the DOT lenses to optimize the efficacy of myopia control while maintaining acuity and tolerability.

**Figure 2.**
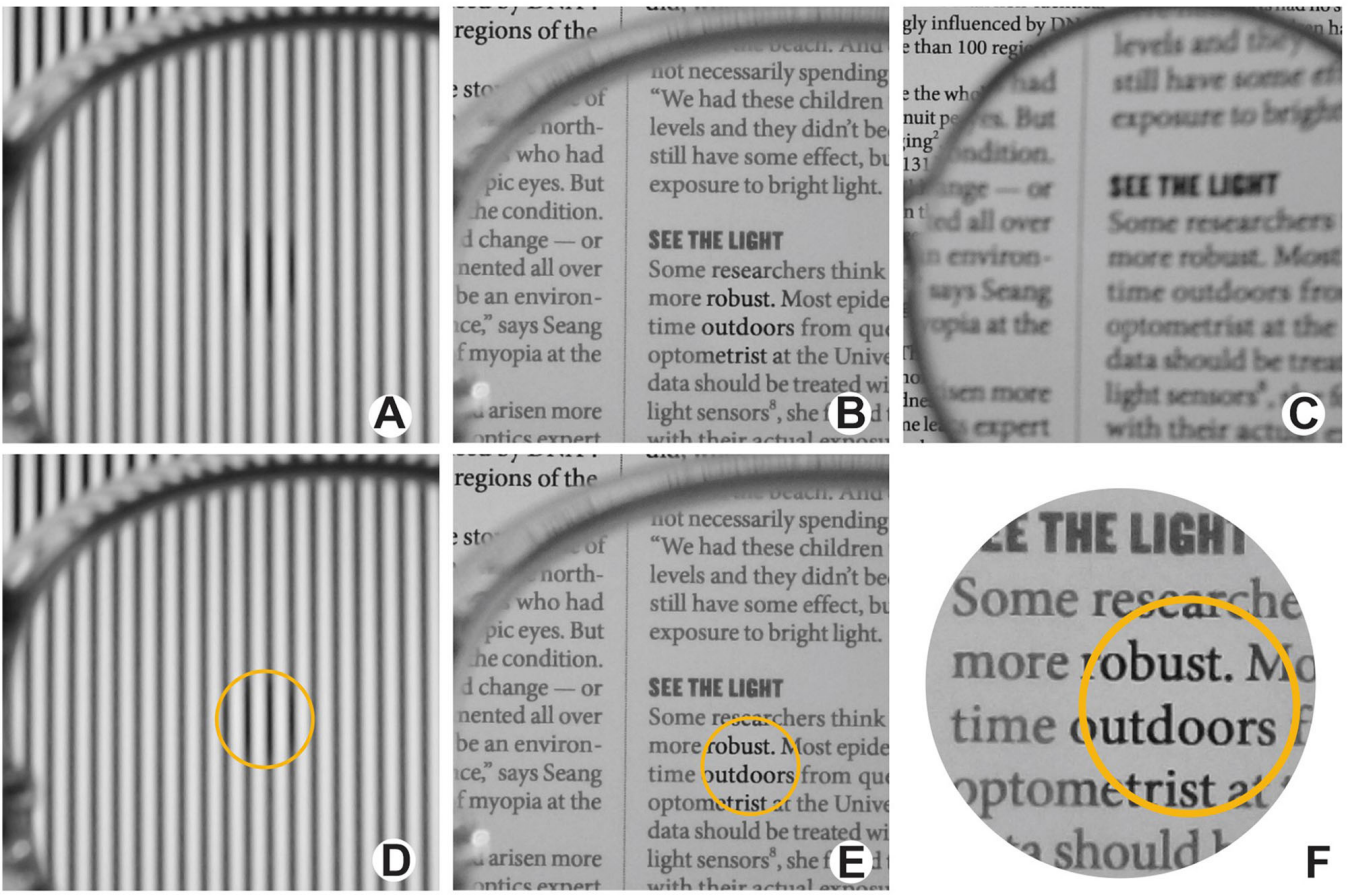
Images photographed through a plano DOT lens (**A**, **B**, **D**, **E**, **F**) compared to a positive lens that produces moderate myopic defocus (**C**). A and D are the same image as B and E, except the clear aperture is circled in *yellow* in D and E for easy comparison with the treatment zone. F shows an *inset* from E enlarged to show detail.

We agree that myopic defocus lenses function as spatial low-pass filters. This well-known phenomenon is demonstrated in Figure 2C, where a positive-power trial lens was used to produce moderate myopic defocus. The loss of high spatial frequencies blurs the text, eliminating the sharp edges making it harder to read. This is similar to the results for the MyoCare 0D lenses, which incorporate cylindrical annular refractive elements, as reported by Gawne et al.^2^ in their letter to the Editor. In contrast, DOT lenses were designed to reduce the amplitude of spatial frequencies where the peripheral midget bipolar cells are most sensitive (Fig. 1) while limiting the reduction of the high spatial frequency components essential for high acuity foveal vision. This is illustrated in the images taken through a DOT spectacle lens (Figs. 2B, 2E, 2F). The DOT lenses are designed with a central clear aperture surrounded by microscopic light-scattering elements. A comparison of the image through the clear aperture compared to the treatment zone allows visualization of the contrast-lowering effects of the lenses. Figures 2B and 2E are the same image, but 2E shows the clear aperture outlined in yellow. From our own experience wearing DOT lenses, these images accurately represent the user experience. Figure 2F is enlarged from an inset from Figure 2E, showing the details of the difference in contrast between the clear aperture and the treatment zone. The light-scattering elements were designed to produce wide-angle scatter, distributing the scattered light as uniformly as possible to produce a uniform reduction in contrast across spatial frequencies. For the images of Figures 2B, 2E, and 2F, the reduction in contrast between the darkest black and lightest white was measured to be 45%, comparing the treatment zone and clear aperture. According to contrast theory, this reduction in contrast is the basis of the effectiveness of the DOT lenses in myopia control.

In the current version of the lenses, there is a greater reduction in contrast at higher spatial frequencies, but the difference is slight compared to myopic defocus (Fig. 2C) and to approaches using positive refractive elements like the MyoCare 0D lenses tested by Gawne et al.^2^ The reduction in contrast at low spatial frequencies is illustrated in Figures 2A and 2B. A one-cyc/deg sine wave grating is imaged through a DOT lens. The reduction in contrast of the grating through the treatment zone was measured to be 28% compared to the clear aperture. Reduction in contrast at this spatial frequency is necessary because, as shown in Figure 1, the peripheral midget bipolar cells are still relatively sensitive at 1 (log 0) cyc/deg, and contrast needs to be reduced for this frequency for optimal myopia control according to contrast theory.

There are two competing explanations for the effectiveness of DOT lenses in slowing myopia progression. Contrast theory holds that DOT lenses work by lowering contrast at spatial frequencies to which peripheral midget bipolar cells are sensitive, while Gawne and colleagues propose that they reduce interfering higher spatial frequency noise while preserving the spatial frequencies in the 3-4 cyc/deg range. These theories lead to very different predictions about the relative effectiveness of two myopia control lens designs: DOT lenses versus MyoCare lenses. Gawne et al.^2^ have shown that because they are based on myopic defocus, the MyoCare lenses preferentially reduce high spatial frequencies similar to what is shown in Figure 2C, whereas the DOT lenses were designed to reduce contrast for the frequencies to which the peripheral midget bipolar cells are sensitive (Fig. 1) while preserving the high spatial frequencies essential for high acuity foveal vision.

It is now possible to test these predictions because one-year myopia control data has recently been published for the MyoCare lenses.^8^ MyoCare lenses had a relative efficacy of 48% for spherical equivalent refraction (SE) and 45% reduction for axial length (AL) compared to single-vision lenses. These values can be compared to the 12-month results for the DOT lenses,^9^ which had a 74% reduction in SE and a 50% reduction in AL. The differences between the results for the two types of lenses must be interpreted cautiously because the trials were performed on different study populations. However, the fact that the DOT lenses performed better than MyoCare OD is consistent with contrast theory, which holds that the lenses work by lowering contrast at spatial frequencies to which peripheral midget bipolar cells are sensitive. Moreover, by relative preservation of high spatial frequencies essential for high-acuity foveal vision, the DOT lenses provide a superior user experience compared to peripheral defocus approaches to myopia control.
